# Cortical hypertrophy with a short, curved uncemented hip stem does not have any clinical impact during early follow-up

**DOI:** 10.1186/s12891-015-0830-9

**Published:** 2015-12-01

**Authors:** Michael W. Maier, Marcus R. Streit, Moritz M. Innmann, Marlis Krüger, Jan Nadorf, J. Philippe Kretzer, Volker Ewerbeck, Tobias Gotterbarm

**Affiliations:** Clinic for Orthopedics and Trauma Surgery, Heidelberg University Hospital, Schlierbacher Landstrasse 200a, 69118 Heidelberg, Germany

**Keywords:** Total hip arthroplasty, Short stem prosthesis, Fitmore® hip stem, Survival rate, Clinical outcome, Radiographic analysis, Cortical hypertrophy

## Abstract

**Background:**

Short stems have become more and more popular for cementless total hip arthroplasty in the past few years. While conventional, uncemented straight stems for primary total hip arthroplasty (THA) have shown high survival rates in the long term, it is not known whether uncemented short stems represent a reasonable alternative. As cortical hypertrophy has been reported for short stems, the aim of this study was to determine the radiographic prevalence of cortical hypertrophy and to assess the clinical outcome of a frequently used short, curved hip stem.

**Methods:**

We retrospectively studied the clinical and radiographic results of our first 100 consecutive THAs (97 patients) using the Fitmore® hip stem. Mean age at the time of index arthroplasty was 59 years (range, 19 – 79 years). Clinical outcome and radiographic results were assessed with a minimum follow-up of 2 years, and Kaplan-Meier survivorship analysis was used to estimate survival for different endpoints.

**Results:**

After a mean follow-up of 3.3 years (range, 2.0 – 4.4 years), two patients (two hips) had died, and three patients (four hips) were lost to follow-up. Kaplan-Meier analysis estimated a survival rate of 100 % at 3.8 years, with revision for any reason as the endpoint. No femoral component showed radiographic signs of loosening. No osteolysis was detected. Cortical hypertrophy was found in 50 hips (63 %), predominantly in Gruen zone 3 and 5. In the cortical hypertrophy group, two patients (two hips; 4 %) reported some thigh pain in combination with pain over the greater trochanter region during physical exercise (UCLA Score 6 and 7). There was no significant difference concerning the clinical outcome between the cortical hypertrophy and no cortical hypertrophy group.

**Conclusions:**

The survival rate and both clinical and the radiographic outcome confirm the encouraging results for short, curved uncemented stems. Postoperative radiographs frequently displayed cortical hypertrophy but it had no significant effect on the clinical outcome in the early follow-up. Further clinical and radiographic follow-up is necessary to detect possible adverse, long-term, clinical effects of cortical hypertrophy.

## Background

Uncemented, straight titanium stems are widely used for total hip arthroplasty (THA) and have shown good to excellent long-term survival rates [1–7]. Based on their design, there are accepted disadvantages of conventional straight stems, such as proximal stress shielding and loss of bone stock, risk of fracture of the greater trochanter, and a challenging implantation procedure by using a minimally invasive approach [8]. Short-stem prostheses with their metaphyseal and short diaphyseal anchoring were developed primarily to preserve femoral bone stock at the greater trochanter and the insertion of the gluteal muscles by reducing the amount of bone removed during femoral preparation [9]. In addition, short-stem prostheses are thought to facilitate possible future revision surgery although so far there is no support in the literature for this assumption.

**Fig. 1:**
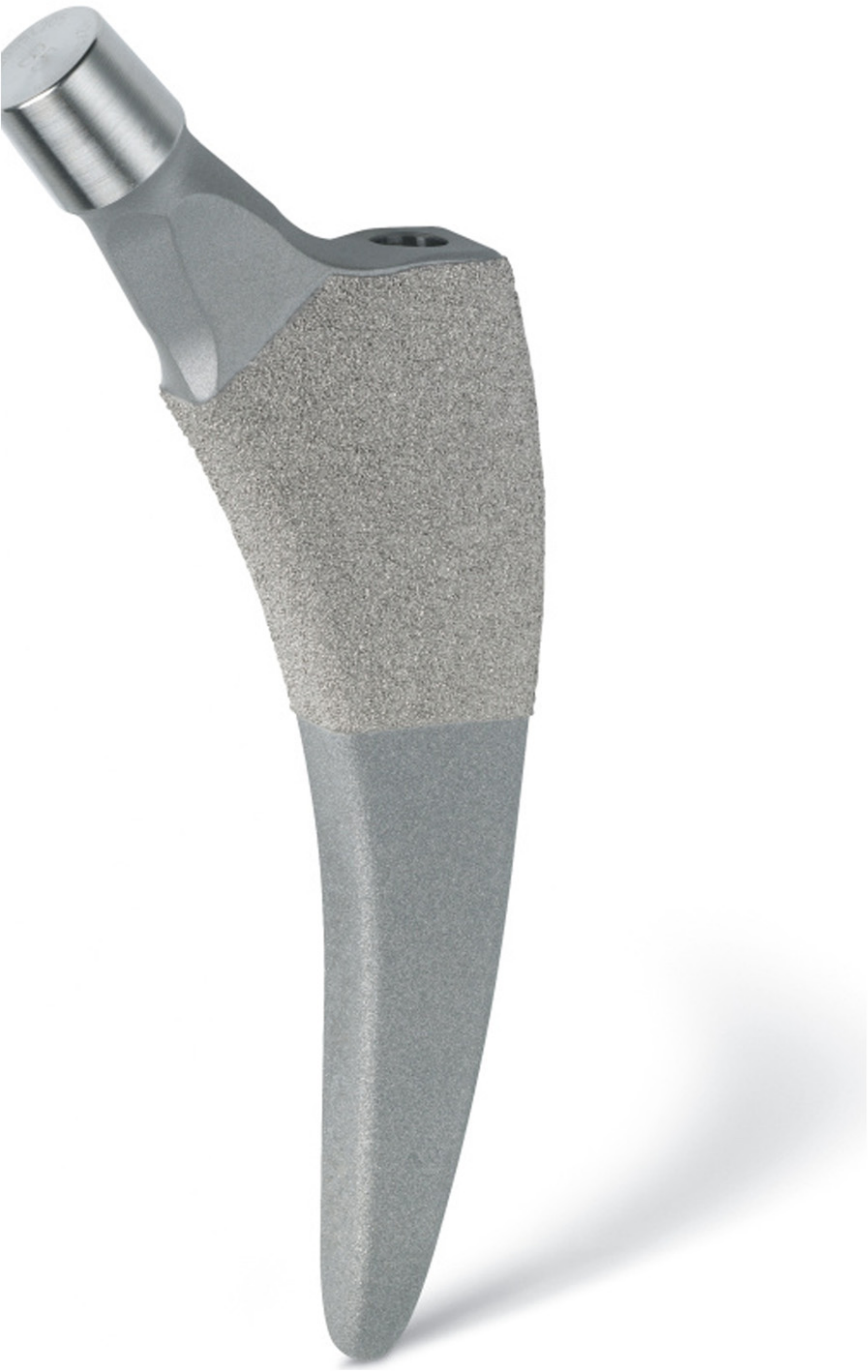
Photograph showing the Fitmore® hip stem as a curved, uncemented, short stem with a trapezoidal cross-section (by friendly permission of Zimmer Biomet)

The Fitmore® hip stem (Zimmer, Warsaw, IN) (Fig. 1) is a curved, uncemented, short-stem prosthesis which was introduced into clinical routine in 2007. Its curved design is thought to transmit load proximally and thus to give an optimal fit in the calcar region. Its primary stability is presumably achieved by press-fit fixation of the triple-tapered design [10]. With its multiple offset options, the stem allows an offset reconstruction independent of stem size and thus can balance soft tissue of the hip individually [8]. To date, only few studies have investigated the clinical and radiographic outcome for short-stem prostheses with a mid- to long-term follow-up. A 10-year follow-up study of the Pipino stem reported an 82 % survival after 10 years [11]. The results of the first 162 Mayo short stems published by Morrey [12] reported revision surgery in 6 % of THAs after a 6-year follow-up. Santori et al. [13], designers of the ultra-short Proxima stem, did not report any instance of femoral revision in a study group of 129 stems after a mean follow-up of 8 years. Gustke [14] published an experience report of 500 Fitmore® hip stems, with a mean follow-up of 1.3 years. He reported a survival rate of 99.4 % with femoral revision for any reason as the endpoint and about 29 % cortical hypertrophy of his first 100 cases. The radiological finding of cortical hypertrophy has been shown in the past to possibly compromise clinical outcome at both short- and long-term follow-up.

Since uncemented straight stems have demonstrated excellent long-term results into the third decade, newly introduced short-stem designs need to be critically evaluated for their clinical and radiographic outcome. The present study is the first to analyze the prevalence of cortical hypertrophy and to assess the clinical outcome of a frequently used short, curved hip stem for primary cementless THA in order to identify any compromising effect during early follow-up.

## Patients and methods

**Fig. 2:**
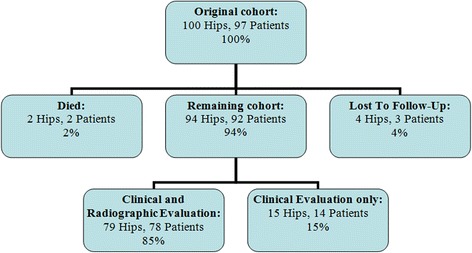
Distribution of hips at final follow-up

From November 2007 to January 2009 a total of 100 consecutive primary THAs (97 patients) were performed at our department, using the Fitmore® hip stem (Zimmer, Warsaw, IN) (Fig. 1). The mean age of the patients was 59 years at the time of index surgery (range, 19–79 years). The mean follow-up was 3.3 years (range, 2.0–4.4 years). During the follow-up period, two patients (two hips) had died with the prosthesis in situ and three patients (four hips) were lost to follow-up, leaving a total of 92 patients (94 hips) for evaluation (Fig. 2). Minimum 2-year radiographic follow-up was available for 79 of the 100 hips (79 %). Patient demographics and diagnoses are listed in Tables 1 and 2. The patients underwent surgery prior to being recruited for the study and all data was collected though retrospective chart review. Informed written consent was obtained from all patients. The study was approved by the ethics committee at the University of Heidelberg, Germany, and it was conducted in accordance with the Declaration of Helsinki of 1975, as revised in 2008.

**Table 1:** Patient demographics

Demographics	Male	Female	Total/mean (range)
Age, mean (range)	56 (23–78)	58 (19–79)	59 (19–79)
Hips	55	45	100

**Table 2:** Patient demographics

Diagnoses	Number of hips (%)
Osteoarthritis	48 (48 %)
Avascular Necrosis	7 (7 %)
Dysplasia Hip	37 (37 %)
Posttraumatic OA	4 (4 %)
Rheumatoid Arthritis	4 (4 %)
Distribution	Hips
Right	42
Left	58

The Fitmore® hip stem (Zimmer, Warsaw, IN) (Fig. 1) is a curved, uncemented, short stem with a trapezoidal cross-section (Fig. 1). The anchorage is mainly metaphyseal, in the intertrochanteric region, and slightly diaphyseal. The primary stability of this short stem is supposed to be achieved by a press-fit fixation of the triple-tapered design [10]. A porolock titanium vacuum plasma spray (Ti-VPS) coating in the proximal part was chosen to enhance bone ongrowth. This short-stem system is comprised of four families A, B, B-extended, and C to respect offset variations [10]. The families were developed to address the relationship between head center and medial curvature of the femoral canal. In this study 95 patients (98 hips) received an Allofit press-fit acetabular component (Zimmer, Winterthur, Switzerland) and two patients (2 hips) a Pinnacle acetabular cup (Depuy, Warsaw, IN). In all patients (100 hips), a 32-mm Al2O3 ceramic head (Biolox forte®; CeramTec, Plochingen, Germany) articulating with highly cross-linked polyethylene (HXLPE) (Durasul®; Zimmer, Winterthur, Switzerland) was used.

All operations were performed at our department by two experienced surgeons. The standard surgical approach was either a modified Watson-Jones [15] or a transgluteal Bauer approach [16] with the patient in supine position. After serial femoral broaching, an intraoperative anteroposterior (AP) radiograph was made with the last broach in situ to show sufficient fit in the coronal plane for optimal press-fit of the femoral component. Full weight-bearing was allowed directly after surgery. For routine prophylaxis of heterotopic ossification, diclofenac 75 mg was administered twice per day for 2 weeks. Intravenous, third-generation cephalosporin (1.5 g cefuroxime) was administered peri-operatively. Anticoagulation therapy consisted of low-molecular-weight heparin administered subcutaneously the day before surgery and continued for 6 weeks postoperatively.

Clinical and functional outcome was assessed by an independent observer pre- and postoperatively, including documentation of thigh pain, Harris Hip Score (HHS) [17], University of California, Los Angeles (UCLA) activity [18], and Tegner score [19–21]. Additionally, patients completed a McMaster Universities Osteoarthritis index (WOMAC) questionnaire postoperatively [22] at final follow-up.

For radiographic evaluation, standard pelvic AP and lateral radiographs of the hip were taken directly postoperatively and at final follow-up. Radiolucent lines or osteolysis at the femoral bone-prosthesis interface as well as cortical hypertrophy were evaluated by two independent, blinded observers (MM, MS) using the zones described by Gruen et al. [23] as previously described by our study group [24]. Femoral osteopenia due to stress shielding and femoral component fixation were graded according to the criteria described by Engh et al. [25, 26]; femoral loosening was defined as progressive axial subsidence of more than 5 mm according to the description of Malchau et al. [27] or varus or valgus tilting. Furthermore, heterotopic ossification was assessed according to Brooker et al. [28].

### Statistical analysis

Kaplan-Meier survivorship analysis was used to estimate survival for different end points: stem revision for any reason, revision for aseptic loosening, and reoperation on the hip for any other cause (e.g., liner exchange, infection, periprosthetic fracture, or dislocation). Revision was defined as an operation that involved removal and/or replacement of one or more components of the cup, inlay, head, or stem. The data were not normally distributed, and Wilcoxon rank-sum test was used to compare pre- and postoperative clinical scores and the postoperative scores between the cortical hypertrophy and no cortical hypertrophy group. We considered p-values of <0.05 to be significant. SPSS® Version 17.0 (SPSS Inc, Chicago, IL, U.S.A.) and Graphpad Prism® Version 5.0 (Graphpad Software, La Jolla, CA, U.S.A.) were used to record and analyze the collected data.

## Results

### Sample

**Fig. 3:**
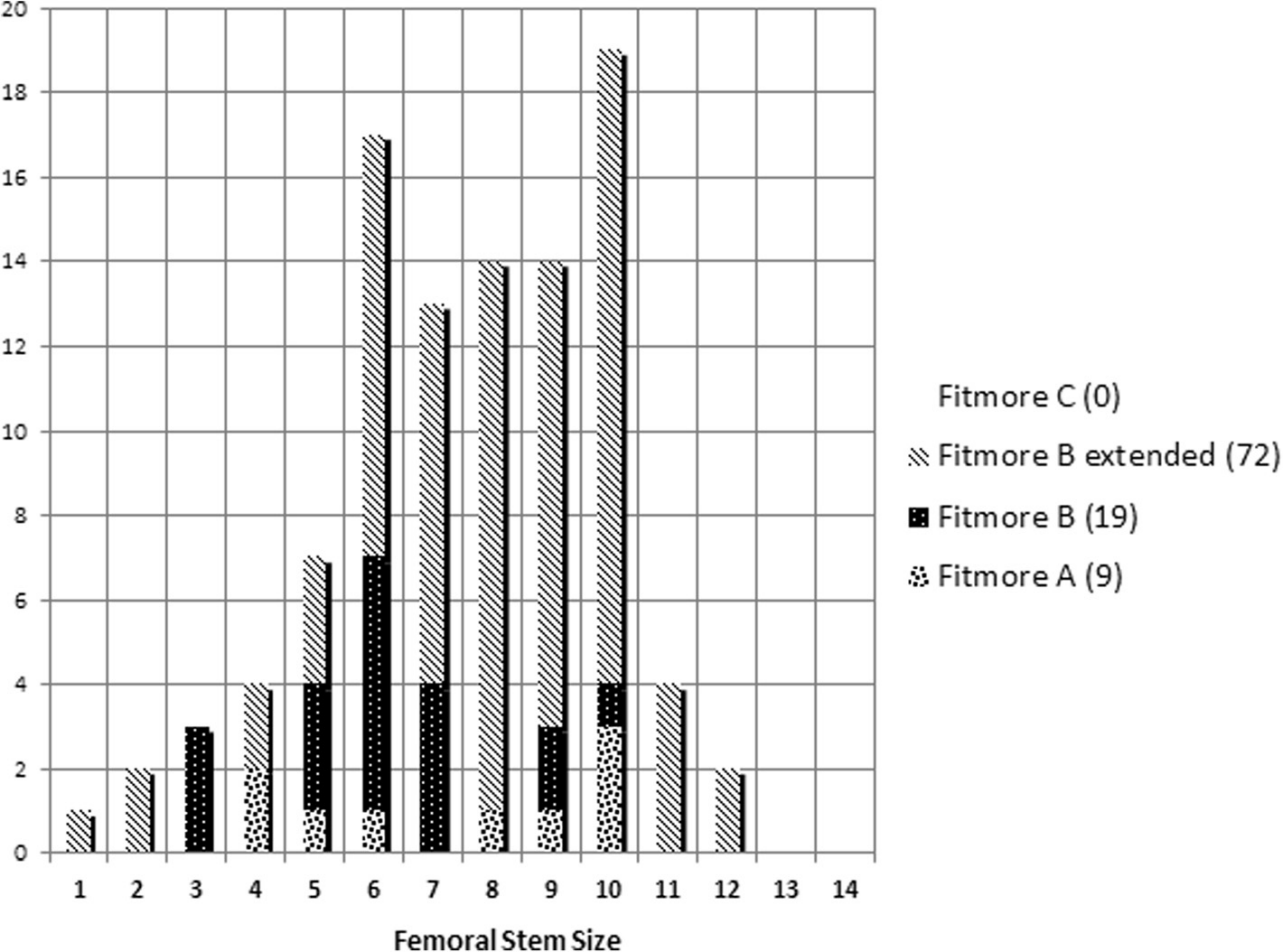
Distribution of the Fitmore® hip stem families and stem sizes

At a minimum follow-up of 2 years, two patients (two hips) had died with the prosthesis in situ, and three patients (four hips) were lost to follow-up as they had moved to foreign countries (Fig. 2). Thus, 94 hips in 92 patients were available for review at a mean follow-up of 3.3 years (range, 2.0–4.4 years). In all, 78 patients (79 hips) were available for both clinical and radiographic evaluation, leaving 14 patients (15 hips) for clinical evaluation without radiographs (Fig. 2). The distribution of our patients to the 4 Fitmore® families A, B, B-extended, and C and the stem sizes is shown in Fig. 3.

### Revisions

Of all 100 THAs there have been no reports (0 %) of femoral stem revisions. In one hip (one patient) a reoperation was performed for hematoma 3 days after index surgery. There were no intraoperative complications, in particular no proximal femoral fracture during insertion of the new, short, curved, uncemented hip stem. No early or late infection was observed, and at final follow-up no isolated revision of the cup or inlay had been performed.

### Survival analysis

**Fig. 4:**
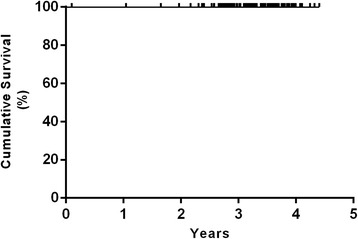
Kaplan-Meier survivorship curve with femoral revision for any reason as the endpoint. The 3.8-year survival was estimated at 100 % (20 hips at risk)

**Fig. 5:**
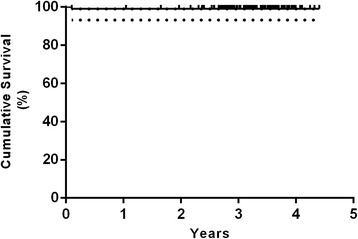
Kaplan-Meier survivorship curve and 95 % CI with reoperation for any reason as the endpoint. The 3.8-year survival was estimated at 99 % (95 %-CI, 93–100 %; 20 hips at risk)

**Fig. 6:**
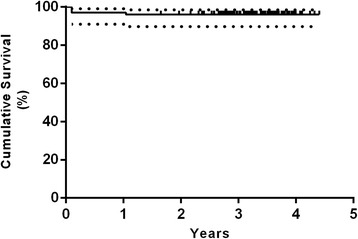
Kaplan-Meier survivorship curve and 95 % CI with worst-case survival of the femoral component (considering all patients that were lost to follow-up as revised). The 3.8-year survival was estimated at 96 % (95 %-CI, 90–98 %; 20 hips at risk)

The Kaplan-Meier survival analysis with femoral revision for any reason as the endpoint estimated a survival rate of 100 % at 3.8 years (20 hips at risk) (Fig. 4). The survival rate with reoperation for any reason as the endpoint was 99 % at 3.8 years (95 % confidence interval [CI], 93–100 %; 20 hips at risk) (Fig. 5). Worst-case survival of the femoral component, considering all patients that were lost to follow-up as revised, was 96 % at 3.8 years (95 % confidence interval [CI], 90–98 %; 20 hips at risk) (Fig. 6).

### Radiographic evaluation

**Fig. 7:**
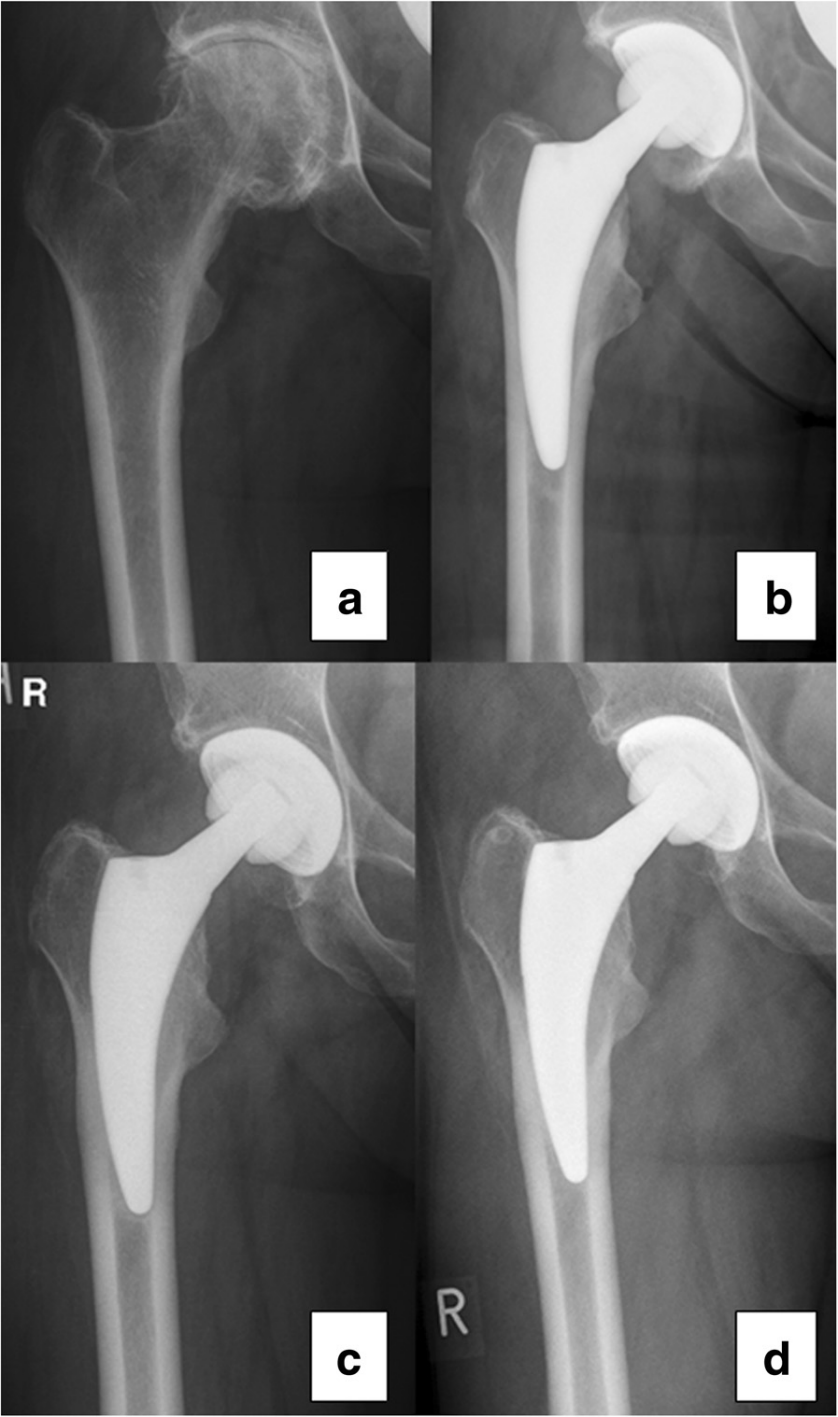
Anteroposterior hip radiographs of a 65-year-old woman with primary OA (**a**), directly postoperatively (**b**), 1 year (**c**), and 4 years (**d**) after surgery. The postoperative follow-up ((**c**) and (**d**)) shows the typical cortical hypertrophy mainly located in Gruen zone 3 and 5 and radiolucency < 2 mm in Gruen zone 1

**Fig. 8:**
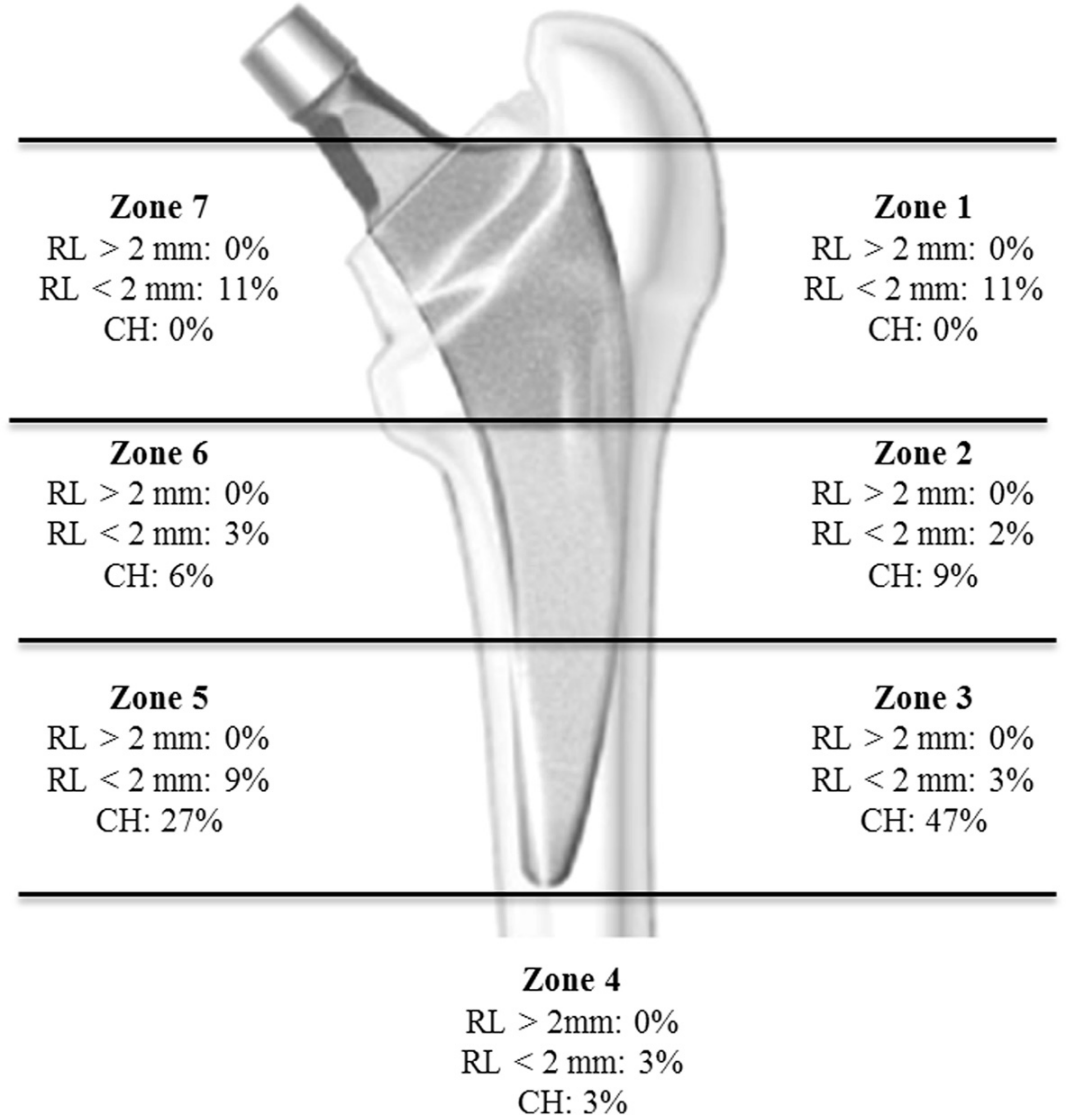
Radiographic evaluation of the radiolucent lines (RL) and cortical hypertrophy (CH) findings in Zones according to Gruen et al

Figure 7 shows a representative AP hip radiograph preoperatively and postoperatively at final follow-up. Radiographic evaluation did not show any acetabular or femoral component loosening at a minimum of 2 years. All implants demonstrated complete osseous ingrowth. No femoral or periacetabular osteolysis or radiolucent lines >2 mm were detected in any hip. Radiolucent lines <2 mm around the femoral component were found in 20 of 79 hips (25 %), predominantly in Gruen zone 1, 5, and 7 (Fig. 8). Cortical hypertrophy was found in 50 hips (63 % of the 79 hips with radiological review), predominantly in Gruen zone 3 and 5 (Fig. 8). Figure 7 shows the cortical hypertrophy typically found in Gruen zone 3 and 5. Thirteen hips (17 %) showed both distal cortical hypertrophy and proximal radiolucent lines (<2 mm). Heterotopic ossification was found in 15 of 79 hips (19 %) and was graded according to Brooker as grade I (12 hips), II (1 hips), and III (2 hips).

### Clinical evaluation

The mean total Harris hip score (HHS) improved significantly from 56 points preoperatively (range, 14 -80 points) to 94 points postoperatively (range, 62 -100 points, paired Student *t*-test, *p* < 0.005). There was no significant difference in postoperative HHS between the cortical hypertrophy (94 points) and no cortical hypertrophy (93 points) group (*p* = 0.846). Two patients (2 %) reported some thigh pain in combination with pain at the greater trochanter during physical exercise (UCLA Score 6 and 7). Both patients demonstrated typical cortical hypertrophy located in Gruen zone 3. The mean preoperative UCLA activity and Tegner score increased significantly from 3.7 (range 2 – 7) preoperatively to 6.7 (range 2 – 10) postoperatively (*p* < 0.005) and from 2.7 (range 1 – 6) to 4.4 (range 2 – 8) (*p* = 0.02), respectively. The mean postoperative WOMAC Score was 93 (range 58.3 – 100). There was no significant difference in postoperative UCLA, Tegner, and WOMAC scores between the cortical hypertrophy and no cortical hypertrophy groups.

## Discussion

Short, curved, femoral stems were designed to preserve femoral bone stock [9, 13]. Preserving the femoral neck provides greater torsional stability and may reduce distal migration of the femoral stem [29]. Metaphyseal femoral loading independent of fixation of the stem may help to reduce resorption of proximal bone due to stress shielding [12, 25, 30]. Currently, short stems have become popular in clinical routine but there are only few reports concerning their clinical and radiographic outcome [9, 11–14, 31–35]. The clinical relevance of the present study is the fact that recently published data have indicated the presence of cortical hypertrophy with the Fitmore® hip stem [14] during early follow-up. The aim of this study, therefore, was to determine the prevalence of cortical hypertrophy in the early follow-up and to assess the clinical outcome of this short stem for primary cementless THA in order to identify any compromising effect on the clinical outcome.

In our study, femoral cortical hypertrophy was observed in 63 % of hips, being mainly located at the distal part of the stem (Gruen zone 3 and 5). There was no significant difference concerning the clinical outcome between the cortical hypertrophy and no cortical hypertrophy group. Two of the cortical hypertrophy patients (4 %) reported some thigh pain in combination with pain at the greater trochanter during physical exercise (UCLA Score 6 and 7). Pipino et al. reported a much higher rate of 14 % persistent thigh pain after 10 years, compared to 4 % in the present series. A similarly low percentage of thigh pain was reported for the uncemented straight titanium Zweymüller stem, showing isolated thigh pain in 1 % of patients after 20 years Thigh pain is known to be related to stem design and implant stiffness [37, 38]. Pipino et al. [11] reported on distal cortical hypertrophy in 48 % of his patients, mainly in Gruen zone 2, 3 and 5. In his long-term follow-up study, he found thigh pain in 14 % directly after surgery, which spontaneously resolved within 1 year. Among his patients with thigh pain, one had an oversized stem with consequential tip wedging, while five had undersized, varus stems. In three of these latter cases, radiographs showed demarcation lines associated with cortical hypertrophy in zones 3 and 5. Pipino et al. interpreted thigh pain to be an expression of transitory instability of the stem, rather than a phenomenon related to tip wedging of the prosthesis. Gustke [14] reported 29 % cortical hypertrophy in his first 100 cases, with only few patients showing distal cortical hypertrophy and proximal cortical atrophy. In the present study, 13 hips showed distal cortical hypertrophy and proximal radiolucent lines (>2 mm) at the same time. Gustke concluded that in these cases, the stem could be fixed more distally. We conclude that these hips need to be reexamined in a shorter follow-up period to detect a potentially higher risk of loosening. In the present study, more than 60 % of the hips showed cortical hypertrophy but it had no significant effect on the clinical outcome in the early follow-up period.

In the present series, the Kaplan-Meier analysis, with femoral revision for any reason as the endpoint, revealed an excellent survival rate of 100 % at 3.8 years. The survival rate with reoperation for any reason as the endpoint was very good at 99 %. In the literature, only one report has been published. Gustke [14] followed up 500 Fitmore® hip stems, with a mean follow-up period of 1.3 years. He reported a survival rate of 99.4 % with femoral revision for any reason as the endpoint, comparing well to our results. However, a comparison with the results of the present study is difficult since distribution of stem types was considerably different and the mean follow-up interval differed as well. Furthermore, Gustke reported two patients who sustained a periprosthetic fracture after falls that subsequently required stem revision. One patient had a late infection. In that series, there were no revisions for aseptic loosening. In the present series, there was no periprosthetic fracture, no infection, and no isolated revisions of the cup or inlay after a mean follow-up of 3.3 years.

In the literature to date, long-term follow-up studies of short stems are rare: There is one long-term follow-up study of the first short stem developed, the Pipino stem, showing an 82 % survival rate at 10 years [11]. The results of the first 162 Mayo short stems published by Morrey [12] reported revision surgery in 6 % of THAs, due to wear-induced loosening. Mechanical failure of the stem resulted in stem revision in three patients (2 %). Intraoperative fracture of the femur occurred in 10 cases (6 %). None of these patients complained of thigh pain. Santori et al. [13], designers of the ultra-short Proxima stem, did not mention any femoral revision in a study group of 129 stems, whereas a total of five hips had to be revised, three for polyethylene liner exchange and two for acetabular loosening [13].

In cementless short-stem prostheses, preserving the femoral neck should provide greater torsional stability and reduce distal migration of the femoral stem [29]. The idea underlying reduced diaphyseal fixation is to achieve proximal load transfer and to reduce stress shielding and thigh pain [9]. In the present study, radiographic evaluation did not show any femoral component loosening, and all implants revealed osseous ingrowth. No femoral osteolysis or radiolucent lines >2 mm were detected; radiolucent lines <2 mm around the femoral component were found in 25 %, predominantly in Gruen zone 1, 5 and 7. Factors related to patients, implant design, and implantation did not predict migration patterns. Bieger et al. [39] compared the Fitmore® hip stem to a conventional straight CLS® stem (Zimmer) in a biomechanical study and showed that the Fitmore® hip stem was as stable as the CLS® stem to tilting in the frontal and sagittal plane and more stable to rotation. The shorter Fitmore® stem showed a lower reduction in the surface strain proximally than the straight stem, indicating that the shorter stem may have less proximal stress shielding. Pepke and Nadorf [40] showed bending results that support the hypothesis that the CLS stem presumably closely follows the bending of the bone, whereas the shorter Fitmore stem acts more rigidly. They concluded that stem rigidity and flexibility need to be considered, as they may influence the load transfer at the implant-bone interface and thus possibly affect bone remodeling processes. One of the original ideas behind the design criteria of short stems was to render loading of the proximal femoral bone as physiological as possible, with the aim to reduce stress shielding. However, cortical hypertrophy in the distal Gruen zones implies distal loading and proximal stress bypass. As a limitation of the present study, we only assessed osteolysis on conventional X-rays, which has been shown to be less reliable [41–43]. Albanes et al. [44] showed in a DEXA (dual-energy absorptiometry) study on six different short-stem designs a bone mineral density (BMD) loss that is larger for some designs than for conventional stems. Calculations with FE (finite element) models for the Nanos short stem predicted a bone loss of 30–69 % in Gruen zones 1 and 7 compared to the normal femur [45]. This was confirmed by Tsao et al. with the Mayo hip [46]. A prospective cohort study on the short CFP stem using RSA (radiostereometric analysis) and DEXA [47] concluded that there was substantial loss in proximal BMD (Gruen zone 7: 31 %) and explained this by distally transmitted forces, which would explain the cortical hypertrophy in this study. This was also seen in a prospective 7-year follow-up with qCT-assisted densitometry in 38 CFP stems which showed cancellous BMD loss of 66 % and cortical BMD loss of 27 %, being progressive until 3 years [48]. Their conclusion was that density changes suggest diaphyseal fixation below the lesser trochanter with high proximal bone loss. In the mid- to long-term follow-up, this may evolve into deficient bone support for short stems which rely on proximal fixation.

Currently, the migration pattern of the Fitmore® stem design is still unknown. It has been suggested that there is an association between early excessive migration and medium- to long-term loosening of prosthetic joints [39–41] and that the outcome could be predicted by measuring early migration. Freeman et al. [40] suggested that any new femoral prosthesis should be monitored on introduction, by measuring its migration rate in a number of patients over a period of 2 years, and that rapidly migrating components should be abandoned forthwith. If RSA is used to measure migration, the increased accuracy and precision increase the predictive power [42]. Therefore, RSA studies with the Fitmore® stem are necessary to assess the migration patterns and to predict long-time survival. Considering the heterogeneous group of differently designed, short hip stems, a direct comparison of the results is difficult. Since uncemented straight stems have demonstrated excellent long-term results into the third decade, the requirements for new short stems are high and every outcome study must be evaluated critically.

The present study has some limitations. We only conducted a radiological investigation of cortical hypertrophy. As evidence from radiological investigations using conventional x-rays are limited without RSA or DEXA measurement, the results need to be interpreted with caution. Retrospective, observational studies have an inherent risk of selection bias, as the ideal patients are selected for these procedures, and of expertise bias, as the operations in this study were performed by hip surgeons with special interest and skills in THA. Furthermore, the conclusions of the study are only restricted to short-term results and as cortical hypertrophy is found frequently, radiographic follow-up is required to detect possible long-term clinical effects of cortical hypertrophy.

## Conclusion

The survival rate and the clinical and the radiographic outcome confirm encouraging results for short, curved, uncemented stems. Postoperative radiographs frequently displayed cortical hypertrophy but it had no significant effect on the clinical outcome at early follow-up. Further clinical and radiographic follow-up and RSA studies are needed to detect possible adverse, long-term clinical effects of cortical hypertrophy.

## Abbreviations

BMD: Bone mineral density
DEXA: Dual-energy absorptiometry
FE: Finite element
HHS: Harris Hip Score
RSA: Radiostereometric analysis
THA: Total hip arthroplasty
UCLA: University of California, Los Angeles activity score
WOMAC: McMaster Universities Osteoarthritis index questionnaire
